# Triage mistakes in the Psychiatry Emergency Room: do we really know how to rule out organicity?

**DOI:** 10.1192/j.eurpsy.2024.1179

**Published:** 2024-08-27

**Authors:** H. Andreu, A. Giménez-Palomo, L. Bueno, E. Cesari, O. De Juan, J. I. Mena, I. Ochandiano, L. Olivier, S. Salmerón, M. Sagué-Vilavella

**Affiliations:** Psychiatry and Psychology Department, Institut Clínic de Neurociències, Hospital Clínic de Barcelona, Barcelona, Spain

## Abstract

**Introduction:**

In Spain and other European countries, patients coming to the emergency room (ER) are usually classified as “organic” or “psychiatric” on arrival, but this may be complicated when psychiatric history is present as the focus can be misplaced (Leeman. IJPM 1975;6(4):544-40; Alam et *al.* Psychiatr. Clin. North Am. 2017;40(3):425–33).

**Objectives:**

To describe three cases seen in the psychiatric emergency room (PER) in which triage errors occurred and to review whether it is widespread for psychiatric patients with organic pathology or in need of medical care to be wrongly triaged.

**Methods:**

We retrospectively reviewed three cases seen in the PER of Hospital Clínic in July 2023 in which triage errors happened. Triage error was considered when patients triaged directly to the PER presented symptoms that either needed medical treatment or required medical clearance before being considered purely psychiatric.

**Results:**

**Case 1:** A 27-year-old woman with history of depressive syndrome was triaged for a speech disturbance that had occurred fifteen minutes after intercourse. After being evaluated, she was referred to neurology where she was diagnosed with an acute ischaemic stroke in left middle cerebral artery territory, requiring thrombectomy and posterior admission to neurology.

**Case 2:** A 50-year-old man with history of alcohol use disorder was brought to the PER after saying that “he was seeing people doing magic” at home. When evaluated, significant distal tremor, tachycardia and hypertension were observed, being compatible with withdrawal symptoms, so he was transferred to the ER. There he was monitored and treated, finally requiring admission to internal medicine due to persistent symptoms.

**Case 3:** A 26-year-old man with history of substance use disorder was triaged for loss of consciousness and “spasms”. After evaluation, he was transferred to the ER, where organic screening was carried out, being oriented as a probable tonic-clonic seizure and discharged with outpatient follow-up.

**Conclusions:**

The cases presented are instances in which somatic diseases in patients pre-labelled with psychiatric histories were wrongly assumed to be recurrences of their psychiatric disorders. In all cases, they needed to be re-examined by the corresponding medical specialty and required diagnostic tests, in two cases hospital admission was needed. Emergency physicians and emergency psychiatrists often disagree on how to medically clear patients (Alam et *al.* Psychiatr. Clin. North Am. 2017;40(3):425–33; Janiak et *al.* JEM. 2012;43(5):866–70), some authors have even proposed protocols for doing this in a more systematic way (Shah et al. JEM. 2012;43(5):871–5). To avoid a delay in diagnosis and treatment and the consequences that may result from it, establishing guidelines for proper triage of patients with psychiatric history should be considered.

**Disclosure of Interest:**

None Declared

